# Bovine tuberculosis prevalence and risk factors in selected districts of Bangladesh

**DOI:** 10.1371/journal.pone.0241717

**Published:** 2020-11-10

**Authors:** S. K. Shaheenur Islam, Tanzida Begum Rumi, S. M. Lutful Kabir, Adri G. M. van der Zanden, Vivek Kapur, A. K. M. Anisur Rahman, Michael P. Ward, Douwe Bakker, Allen G. Ross, Zeaur Rahim

**Affiliations:** 1 Department of Microbiology and Hygiene, Bangladesh Agricultural University, Mymensingh, Bangladesh; 2 Department of Livestock Services, Ministry of Fisheries and Livestock, Dhaka, Bangladesh; 3 International Center for Diarrhoeal Disease Research, Dhaka, Bangladesh; 4 LAB MICTA, Hengelo, The Netherlands; 5 Department of Animal Science, The Pennsylvania State University, University Park, Pennsylvania, United States of America; 6 Department of Medicine, Bangladesh Agricultural University, Mymensingh, Bangladesh; 7 Sydney School of Veterinary Science, The University of Sydney, Camden, New South Wales, Australia; 8 Independent Expert, Lelystad, The Netherlands; University of Lincoln, UNITED KINGDOM

## Abstract

A cross-sectional survey was conducted in selected districts of Bangladesh to estimate the prevalence of bovine tuberculosis (bTB), and to identify the risk factors for bTB. We included 1865 farmed cattle from 79 herds randomly selected from five districts. Herd and animal level data were collected using semi-structured interviews with cattle herd owners. The single intradermal comparative tuberculin test (SICTT) was used to estimate the prevalence of bTB. The risk factors were identified using mixed-effect multiple logistic regression analyses. The overall herd and animal level prevalences of bTB were estimated to be 45.6% (95% Confidence Interval [CI] = 34.3–57.2%) and 11.3 (95% CI = 9.9–12.8%), respectively, using the OIE recommended >4 mm cut-off. The true animal level prevalence of bTB was estimated to be 11.8 (95% Credible Interval = 2.1–20.3%). At the herd level, farm size, bTB history of the farm and type of husbandry were significantly associated with bTB status in univariable analysis. Similarly, age group, sex, pregnancy status and parity were significantly associated with bTB at cattle level. However, in multivariable analysis only herd size at the herd level and age group and pregnancy status at the cattle level were significant. Compared to a herd size of 1–10, the odds of bTB were 22.8 (95% CI: 5.2–100.9) and 45.6 times (95% CI: 5.0–417.7) greater in herd sizes of >20–50 and >50, respectively. The odds of bTB were 2.2 (95% CI: 1.0–4.5) and 2.5 times (95% CI: 1.1–5.4) higher in cattle aged >3–6 years and > 6 years, compared to cattle aged ≤1 year. Pregnancy increased the odds of bTB by 1.7 times (95% CI: 1.2–2.4) compared to non-pregnant cattle. Taken together, the results suggest high herd and animal level prevalence of bTB in these 5 districts, with the greatest risk of bTB in older and pregnant cattle within large herds (>20), and highlight an urgent need for continued surveillance and implementation of bTB control programs in Bangladesh.

## Introduction

Bovine tuberculosis (bTB) is a bacterial disease of cattle mainly caused by *Mycobacterium bovis*, *a member of M*. *tuberculosis complex*. [1]. However, *M*. *orygis* has been reported to be the main causative agent of bTB in Bangladesh [2, 3]. Cattle are the primary hosts for *M*. *bovis*, but other captive wild mammals (including deer, monkey, giraffe and wildebeest) as well as humans can also be infected. The disease can occur as subacute or chronic forms, with a variable degree of progression. A small number of animals may show clinical signs within a few months of infection while others may require several years to develop clinical signs. In low and middle income countries (LMICs), bTB is still common and it induces severe economic losses that can occur from livestock deaths, chronic disease, and reduced production. For example, in Ethiopia the economic cost of bovine TB was US$75.2 million and US$358 million in 2005 and 2011, respectively [4]. Moreover, the disease affects the health and livelihood of already marginalized small and backyard livestock owners as well as consumers of milk and dairy products in general [5].

Globally, it was estimated that 143,000 (95% CI = 71,200–240,000) cases and 12,300 (95% CI = 4,820–23,300) deaths due to zoonotic tuberculosis (zTB) caused by *M*. *bovis* occurred in 2018. Bangladesh is located in the WHO’s South East Asian region where the total regional burden of zTB is 44,800 (11,500–100,000) and an estimated number of deaths of 2,090 (95% CI = 571–4,620) [6]. It has been estimated that *M*. *bovis* accounts for 3.1% of all human TB cases, 2.1% of all pulmonary and 9.4% of all extra-pulmonary TB (EPTB) cases [7]. Of the overall TB incidence rate (≤71/100,000 population), ≤1.4% is linked to zTB [8]. However, the contribution of bovine tuberculosis to the national tuberculosis burden in Bangladesh remains unknown [9] and has always been neglected.

Bangladesh is routinely notifying the presence of bTB in cattle as *M*. *tuberculosis* complex (MTC) to the OIE. However, the actual bTB burden is most likely to be underestimated due to a lack of active surveillance in Bangladesh. Several studies have been conducted in the past to estimate the prevalence of bTB in different geographical locations of Bangladesh. Pharo et al. (1981) reported 6.0% tuberculin skin test (TST) positive cattle in Sirajgonj district [10]. Samad and Rahman (1986) reported 3.0% TST positive cattle in dairy farms of Bangladesh Agricultural University, Mymensingh and Government Dairy farm of Sylhet and 2.0% TST positive cattle in non-organized rural cattle in Mymensingh, Tangail and Rajshahi districts. Another study confirmed 27.7% prevalence in breeding bulls (n = 137) [11]. The prevalence of bTB were also reported to be 3.3% (n = 696) based on caudal fold testing in cattle from several districts [12] and 7.78% (21/270) by rapid tuberculin test kits in cattle of Sirajganj district [13]. However, in most of the previous studies testing was conducted at a less sensitive inoculation site (caudal fold in tail) without adjusting the doses of this purified protein derivatives (PPDs) to be administered [5] and a few studies used rapid test kits that are not OIE approved and presumed to be less sensitive as well [13, 14]. Eighty five percent (85%) of the national cattle populations consist of non-descript indigenous cattle and 15% are crossbred cattle [15]. Indigenous cattle are less productive and unable to meet the national demand for milk and meat. To enhance the productivity of indigenous cattle, cross breeding with exotic breeds (mostly Holstein Friesian) has been ongoing since the 1980s. As a result the number of cross-breed cattle are increasing gradually in Bangladesh [16]. These practices likely increase the burden of bTB because cross-breed cattle are hypothesized to be more susceptible to bTB than indigenous cattle [17]. Moreover, identification of the risk factors associated with the occurrence of bTB at herd and animal levels are needed to develop fit-for-purpose and effective control programs. Hence, our study aims were to estimate the prevalence of bTB at the herd and animal levels, and identify risk factors for bTB in selected dairy-intensive districts of Bangladesh.

## Materials and method

### Ethical approval

The present study is part of the research protocol No. PR-17121, which was approved by the Research Review Committee (RRC) and Ethical Review Committee (ERC) of the International Center for Diarrhoeal Disease and Research (icddr,b), Bangladesh and by the Animal Welfare and Experimentation Ethical Committee (AWEEC) of Bangladesh Agricultural University (AWEEC/BAU/2019/24). Both written and oral consent were taken from the owner/manager of the cattle farm before conducting tuberculin skin test and data collection.

### Study area

**Fig 1:**
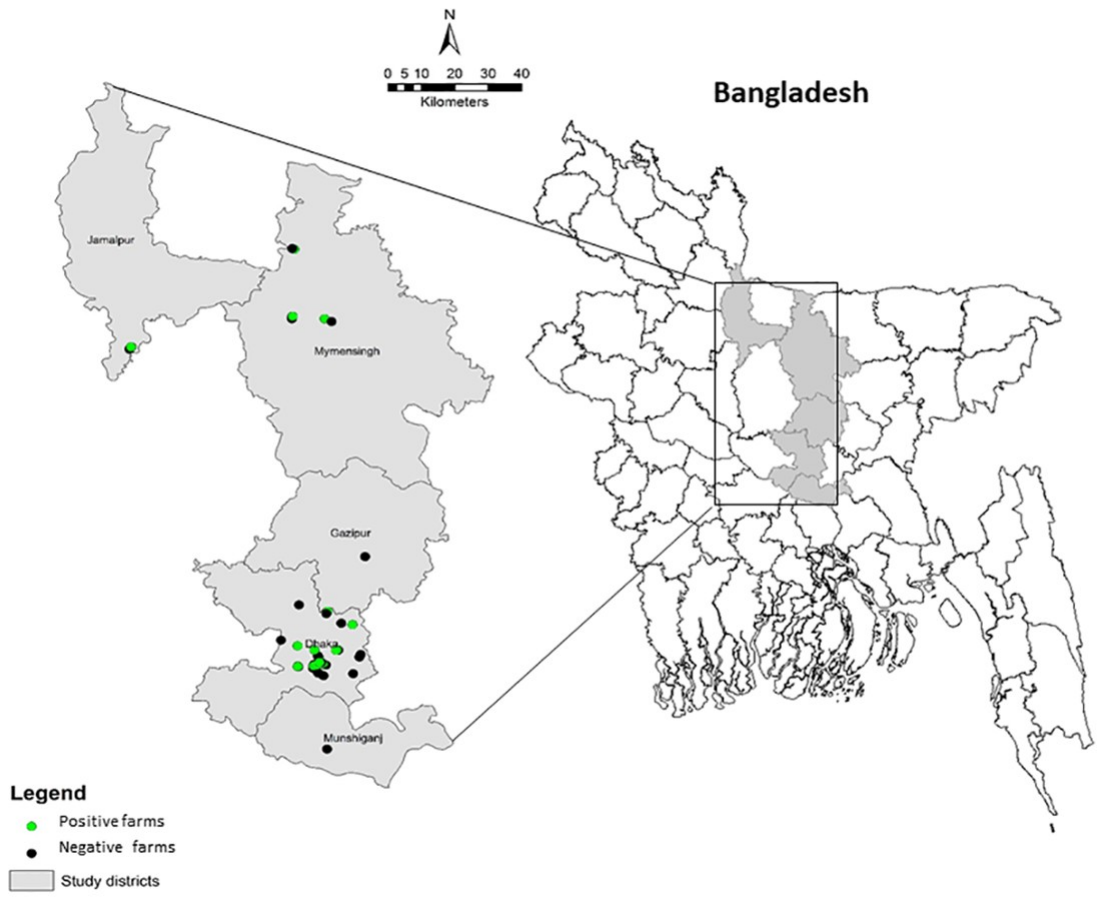
Map of the study districts of Bangladesh. A total of 79 cattle farms of 5 districts were surveyed (as coordinates of some of the farms are closely located, all farms are not pictured separately in the map).

The study was conducted in five dairy intensive districts of Bangladesh viz: Dhaka, Munshiganj, Mymensingh, Gazipur and Jamalpur (Fig 1) from June to December 2019. Geographic coordinates of each dairy farm were captured during tuberculin skin testing by use of a hand-held global positioning system reader (Garmin eTrex 10). ArcGIS-ArcMap version 10.3 (Environmental System Research Institute, Redlands, CA, USA) was used to visualize the spatial distribution of the cattle farms included in this study.

Among the five districts, Dhaka (23°81’N, 90°41’ E), Gazipur (23°7’N, 90°41’ E) and Munshiganj (23°49’N, 90°38’E) districts are located in the central part of the country under Dhaka division, whereas Mymensingh (24°74’N, 90°40’E) and Jamalpur (24°92’N, 89°94’E) districts are located in north-eastern part of the country under Mymensingh division (Fig 1). There are approximately 226 000, 322 000, 102 000, 923 000 and 524,000 heads of cattle in Dhaka, Gazipur, Munshiganj, Mymensingh and Jamalpur districts respectively [18]. Increasing demands for animal origin food, a high density cattle population, very high potential for productivity enhancement, agro-ecological conditions conducive to feed production, accessibility of crop residues, and a choice of mixed crop-livestock farming make these district promising for crossbred cattle farming [19].

### Selection of cattle and farms

The list of dairy farms were obtained from sub-district (Upazila) livestock offices posted in respective district. The list of farms (sampling frame) from five dairy intensive districts of Bangladesh were entered into a spreadsheet (Microsoft Excel 2010). Each farm was assigned with an Excel generated random number using “rand” function. Then the herds were randomly selected from the sampling frame. These districts were: Dhaka, Munshignaj, Mymensingh, Gazipur, and Jamalpur (Fig 1). The farms with ≥2 cattle and at least 2 mature cattle were considered as an inclusion criteria for this study. All animals in a farm were included except calves less than 6 months, advanced pregnant (>8 month), as well as weak and emaciated animals.

### Calculation of sample size and sampling procedure

The sample size was calculated using the formula given in Eq (1) [20]

$${\left(\frac{Z}{d}\right)}^{2}\times [\frac{\left\{\left(S_{e}\times P_{exp})+(1-S_{p})\times (1-P_{exp}\right)\}\times \{\left(1-S_{e}\times P_{exp})-(1-S_{p})\times (1-P_{exp}\right)\right\}}{{\left(S_{e}+S_{p}-1\right)}^{2}}]$$ (1)

Where, *Z* = *Z*—score at 95% confidence interval = 1.96, *S*_*e*_ = average sensitivity of the SICTT = 0.88, *S*_*p*_ = Specificity of SICTT = 0.84 [21], *P*_*exp*_ = expected prevalence = 5% = 0.05 and *d* = level of precision = 5% = 0.05. These assumptions produced a sample size of 468. As cluster sampling was used, the design effect (*D*) of the study was calculated using the formula given in Eq (2) [22].

D=1+(b−1)ρ. (2)

Where *b* the average is number of samples per cluster (15) and *ρ* is the intra-cluster correlation coefficient. The intra-cluster correlation coefficient for bTB is considered to be 0.2 [23]. The design effect was therefore calculated to be *D* = 3.8; when multiplied by the calculated sample size, the minimum sample size then becomes 1778. We assumed average herd size of 15 and hence 119 farms to be tested to reach target sample size. As the herd size varied we needed to visit only 79 herds to reach a sample size of 1865.

### bTB screening by Single Intradermal Comparative Tuberculin Test (SICTT)

The intradermal test (SICTT) was performed as per standard procedure [5, 24]. In this study, avian and bovine purified protein derivatives (PPD) were purchased from Prionics Leylastad BV, Leylastad, The Netherlands. Briefly, two 6–8 square cm areas of skin of selected cattle were shaved 12 cm apart on the left cervical region on day 0. Skin thickness of both these shaved areas was measured using standard slide calipers. Using separate calibrated Mclintock syringes, 0.1 ml bovine PPD (3000 IU/ ml) (Lot. 170506) and 0.1 ml (2500 IU/ ml) (Lot. 171701) avian PPD were injected intradermally in the respective shaved areas. Skin thickness was again measured at both sites at 72±6 h after the injection.

### Risk factor data collection

Information on herd level risk factors was collected from farm owners/managers using a semi-structured and pre-tested questionnaire. The questionnaire contained open-ended and closed-questions and was completed at the time of tuberculin testing. The questions in the questionnaire were translated into the local dialect so that the respondents could easily understand them. The objectives of the survey were explained to each farm owner/farm manager and they could withdraw from the study at any time.

Herd level data on farm type, age of farm, farm size, husbandry type, manure use, feeding of silage, introduction of new animals within last two years, veterinary healthcare provider, and biosecurity status and animal level data on breed, age, sex, parity, weight, milk production, pregnancy status, and body condition score were collected using semi-structured face to face interviews with cattle herders and herd owners at the day 0 of PPDs inoculation (S1 Table, S1 and S2 Questionnaires). Similarly, animal level data collected included sex, age, breed, weight (kg), body condition score (BCS), pregnancy status, milk production, lactation stage and parity (S2 Table, S3 and S4 Questionnaires).

### bTB case definition at animal and herd level

As per the standard criteria of OIE and European Commission [5, 24], if any cattle in a herd is found to be positive in SICTT then that animal and the herd is considered positive for bTB. An animal was considered to be a reactor if the increase in skin thickness at the bovine site of injection was > 4 mm greater than the increase in skin thickness at the avian site of injection. An animal was considered inconclusive if the increase in skin thickness at the bovine site of injection was 2 to ≤ 4 mm greater than the reaction at the site of the avian injection. Similarly when the increase in skin thickness at the bovine site of injection <2 mm, then the animal was considered to be negative.

### Data management and statistical analyses

Animal and farm level data were entered into a spreadsheet (Microsoft Excel 2010). The dataset was coded, checked for integrity and exported to STATA 13 (USA, StataCrop, 4905, Lakeway Drive, College station, Texas 77845,) and R 3.6.0 [25] for analysis.

We summarized the data using descriptive statistics for demographic characteristics and factors. We calculated the mean and standard deviation (SD) for continuous variables and calculated proportions and frequency distributions for categorical variables. All continuous predictor variables (herd size, age of the animal, parity and weight) were categorized prior to logistic regression analysis. Based on the average sensitivity and specificity of the SICTT [21] the animal level true prevalence of bTB was estimated using a Bayesian model described previously [26]. Beta distributions for the priors on sensitivity and specificity of SICTT were calculated using the ‘findbeta’ functions of the package ‘PriorGen’ [27] in R 3.6.0. [25]. The model was run in OpenBUGS [28] with a burn-in period of 50, 000 iterations and estimates were based on a further 50, 000 iterations using three chains. The convergence of the model was assessed by time-series plots, Gelman Rubin convergence diagnostics, autocorrelation plots and Monte Carlo standard errors [29]. The OpenBUGS code used to estimate the true prevalence of bTB is attached as S1 File.

#### Univariable mixed-effect logistic regression analyses

Initially, univariable mixed effects logistic regression analyses were performed by including herd and district as random intercepts for animal and herd level, respectively (R package “lme4” [30]. We used bTB status as the response and each risk indicator variable in turn as an explanatory variable in the model. Any explanatory variable associated with bTB status with a p-value of ≤ 0.10 was selected for multiple mixed-effect logistic regression analysis. Collinearity among explanatory variables was assessed by Cramer’s phi-prime statistic (R package “vcd,” “assocstats” function [31]. A pair of variables was considered collinear if Cramer’s phi-prime statistic was >0.70 [32].

#### Multivariable mixed-effect logistic regression analyses

Manual forward mixed-effect multiple logistic regression analyses were performed to identify risk factors for bovine tuberculosis at animal and herd levels. The best univariate model was selected based on the lowest Akaike’s information criterion (AIC) value. Then the remaining variables were added in turn, based on AIC. The final model selected also had the lowest AIC. Confounding was checked by observing the change in the estimated coefficients of the variables that remained in the final model by adding a non-selected variable to the model. If the inclusion of this non-significant variable led to a change of more than 25% of any parameter estimate, that variable was considered to be a confounder and retained in the model [33]. The two-way interactions of all variables remaining in the final model were assessed for significance based on AIC values, rather than significance of individual interaction coefficients [33]. The intraclass correlation coefficient (ICC), which is a measure of the degree of clustering of bTB positive cattle belonging to the same herd/district, was estimated using the formula:

ICCHerd/District=∂Herd/Ditrict2/(∂Herd/District2+π23).

The 95% confidence interval of the ICC was bootstrapped using the “bootMer” function of the R package “lme4” [30]. All of the above analyses were performed in R 3.6.0 [25].

## Results

### Descriptive epidemiology

SICTT was performed on 1865 cattle from 5 districts. The study included 79 randomly selected dairy farms with a median (interquartile range, IQR) herd size of 11 (6–36). Most farms were in Dhaka (67.0%), followed by Mymensingh (16.5%), Jamalpur (14.0%), Munshiganj (1.3%) and Gazipur districts (1.2%) (Fig 1). Nearly, half of the farms (45.6%) were found to have been involved in cattle farming for > 10 years. About 49% (n = 39) of farms had <10 cattle in their herds. About 57% (n = 45) of farmers kept both dairy and beef cattle and 68.4% (n = 54) of farms were practicing intensive husbandry. More than 90% (n = 77) of farms used fresh cow dung directly in the agricultural field or fish farm without treatment in biogas, and only 6% (n = 5) of farmers provided silage as a cattle feed. Approximately two-third of the farmers (63.3%) maintained a moderate level of biosecurity in their farm and 22.8% (n = 18) of farms kept other animals including sheep, goats and poultry with cattle (mixed farming). Only 9% (n = 7) of farms confirmed that their cattle were infected with bTB previously (Table 1).

**Table 1:** Characteristics of herd composition and management practices (N = 79 cattle farm).

Factor	Category	Frequency number (n)	%
Herd status	Positive	36	45.6
	Negative	43	54.4
History TB in the farm	Yes	7	8.9
	No	51	64.5
	Don’t know	21	26.6
Distribution dairy farms	Dhaka	53	67.0
	Gazipur	1	1.3
	Jamalpur	11	14.0
	Munshiganj	1	1.3
	Mymensingh	13	16.5
Herd size(nos of cattle)	2–10	39	49.4
	10–20	11	13.9
	20–50	18	22.8
	>50	11	13.9
Type of farm	Both (Fattening and Dairy)	45	57.0
	Dairy	34	43.0
Husbandry type	Intensive	54	68.4
	Semi-intensive	25	31.7
Manure use	Use after production of biogas	7	8.9
	Use without biogas	72	91.1
Silage feeding	Yes	5	6.3
	No	74	93.7
Introduction of new animal	No	44	55.7
	Single	12	15.2
	Multiple	23	29.1
Veterinary practitioner	Paraprofessional/ Quack	57	72.2
	Vet	22	27.9
Other animal (sheep, goat, poultry) keeping	Yes	18	22.8
	No	61	77.2
Bio-security type	High	15	19.0
	Low	14	17.7
	Medium	50	63.3

The distribution of key demographics of the cattle tested are shown in Table 2. More than 82.0% (n = 1541) of cattle were Holstein Friesian crossbred (with indigenous). The majority (83.6%) of the tested cattle were female. The median (IQR) age of cattle was 3.7 (1.5–5) years. Around 60% (n = 1071) of cattle were born on the farm. The median (IQR) body weight of the cattle was 380 (200–450) Kg. Out of 1331 cows, 72.7% (n = 967) were milking cows. Among these, 54.5% (n = 527) had calved between 2 and 5 times. Of 1420 female cattle (heifer and cows), approximately 50% (n = 709) were found to be pregnant (Table 2).

**Table 2:** Status of animal level parameters in 1865 cattle in five districts of Bangladesh.

Parameters	Animal (N = 1865) n (N)	%
**SICTT**		
Positive	210 (1865)	11.3
Negative	1523 (1865)	81.7
Inconclusive	132 (1865)	7.1
**Sex of animal**		
Male	305 (1865)	16.4
Female	1560 (1865)	83.6
**Age group**		
≤1 year	284 (1865)	15.2
1–3 years	666 (1865)	35.7
3–6 years	458 (1865)	24.6
More than 6 years	457 (1865)	24.5
**Source of animal**		
Bought	794 (1865)	42.6
Farm	1071(1865)	57.4
**Breed**		
Holstein Friesian cross	1541 (1865)	82.6
Local	117 (1865)	6.3
Other cross (Jersey/Brahman)	83 (1865)	4.5
Shahiwal cross	124 (1865)	6.7
**Weight in Kg**		
1–100	211 (1865)	11.3
100–200	287 (1865)	15.4
200–400	834 (1865)	44.7
400–500	458 (1865)	24.6
>500	75 (1865)	4
**Milking status**		
Yes	967 (1331)	72.7
No	364 (1331)	27.3
**Pregnancy status**		
Yes	708 (1420)	49.9
No	712 (1420)	50.10
**Parity**		
1–2	346 (967)	35.8
3–5	527 (967)	54.5
5+	94 (967)	9.8
**BCS**		
Poor (BCS:0–3)	48 (1865)	2.6
Good (BCS:>6)	1239 (1865)	66.4
Medium (BCS:4–6)	578 (1865)	31

**Fig 2:**
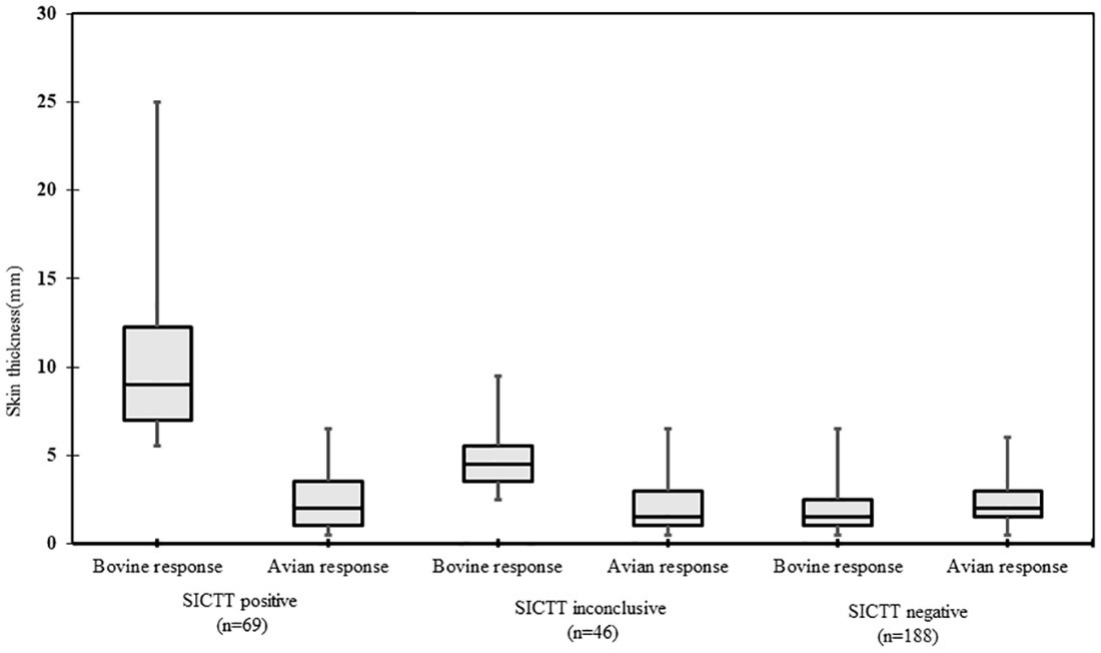
Skin responses at both sites (bovine and avian) of cross reaction of bTB with paratuberculosis and or environmental mycobacterium were documented in 303 (N) cattle in the SICTT. As per standard criteria 69 (>4mm), 46 (2-4mm) and 188 (<2mm) were interpreted as positive, inconclusive and negative (n = 188, <2 mm) animals [5, 24].

Of 1865 cattle tested, 16.3% (n = 303) demonstrated a measurable increase in skin thickness after 72 h of the bovine and avian PPD inoculation both at bovine and the avian sites respectively which reflects cross reaction of bTB with paratuberculosis and or environmental mycobacterium. Of these 303 cattle, 22.8% (n = 69), 15.2% (n = 46) and 62% (n = 188) were interpreted as positive, inconclusive and negative animals respectively (Fig 2 and S3 Table).

### Prevalence of bTB at herd and animal levels

As a whole, herd level prevalence of bTB was 45.6% (95% CI: 34.3–57.2%) (Table 1). The within-herd bTB prevalence ranged from 0–69.2% with an average of 7.5%. The overall cattle level bTB prevalence was estimated to be 11.3% (210 of 1865 individuals; 95% CI: 9.9–12.8) (Table 3). The true animal level prevalence of bTB was estimated to be 11.8 (95% Credible Interval: 2.1–20.3).

**Table 3:** Status of SICTT herds by district, subdistrict/ city corporation and study sites level or using the standard method (positive: >4 mm cut off value, inconclusive = 2–4 mm and negative <2 mm) of interpretation.

District	Subdistrict/City Corporation area	Study area	Inconclusive (2–4 mm)	Negative (<2mm)	Positive (>4 mm cut off value)	Total animal	Farm	Within herd prevalence (%)
Dhaka	DNCC	Demra	3	19	18	40	1	45.0
		Dhour	3	63	5	71	1	7.0
		Rajabari	0	8	0	8	1	-
		Harirampur	2	63	1	66	1	1.5
	DSCC	Meradia	6	41	4	51	1	7.8
		Jamun	16	56	7	79	2	8.9
		Prembag	2	6	1	9	1	11.1
		Mogardiya	20	158	22	200	2	11.0
		Vangamosjid	0	5	0	5	1	-
		Lohargate	10	58	58	126	2	46.0
		Pilkhana	11	116	15	142	3	10.6
		Hazaribag	2	97	1	100	9	1.0
	Dhmarai	Borakoi	4	36	1	41	1	2.4
	Savar	Kathgora	2	44	1	47	1	2.1
		Ragamatiya	1	5	0	6	1	-
		Nagorchor	6	16	11	33	1	33.3
	Keranigang	Barirgoan	2	108	1	111	13	0.9
		Ghaterchar	0	57	3	60	4	5.0
		Atipara	7	35	12	54	2	22.2
		Shikaritola	2	49	0	51	1	-
		Nagda	0	38	1	39	1	2.6
		Brahamankirton	10	30	15	55	3	27.3
	Total		109	1108	177	1394	53	12.7
Munshiganj	Lohagong	Khidirpur	6	30	13	49	1	26.5
	Total		6	30	13	49	1	26.5
Gazipur	Sreepur	Faugan	1	81	1	83	1	1.2
	Total		1	81	1	83	1	1.2
Mymensingh	Sadar	Batta	0	46	1	47	2	2.1
	Sadar	Aqua bypass	1	62	1	64	1	1.6
	Muktagacha	Duttapara	6	50	11	67	3	16.4
	Digorgoan	Muktagacha	5	25	2	32	5	6.3
	Fulpur	Fulpur	0	50	3	53	2	5.7
	Total		12	233	18	263	13	6.8
Jamalpur	Sarishabari	Natherpara	2	55	0	57	6	-
		Tikrapara	2	16	1	19	5	5.3
	Total		4	71	1	76	11	1.3
	Grand total		132	1523	210	1865	79	11.3
	Overall prevalence		7.1 (1865)		11.3 (1865)			

DNCC = Dhaka North City Corporation, DSCC = Dhaka South City Corporation.

### bTB risk factors at cattle and herd level

The herd size, husbandry type and history of bTB in the herd were significantly (P < 0.05) associated in univariable logistic regression analyses (Table 4). At the cattle level, age, sex, pregnancy status and parity were significantly (P < 0.05) associated with bTB status (Table 5). The age group and parity were collinear (Cramer’s phi-prime statistic >0.70) and parity was excluded from the multivariable logistic regression analysis. The history of bTB in a herd was not included in the multiple logistic regression analysis due to missing values.

**Table 4:** Result from univariable logistic regression analysis displaying the relationship between bTB and herd level exposure variables in 79 cattle farms.

Exposure variables	Category	TST positive n (%)	Odds ratio (95% CI)	P value
Age of farm	1–5 years	9 (42.9)	2.8 (0.6–13.2)	0.20
	5–10 years	7 (31.8)	1.0	
	>10 years	20 (55.6)	3.3 (0.9–12.5)	
Herd size	2–10	7 (17.9)	1.0	<0.001
	>10–20	4 (36.4)	2.6 (0.6–11.4)	
	>20–50	15 (83.3)	22.8 (5.2–100.9)	
	>50	10 (90.9)	45.7 (5.0–417.7)	
Type of farm	Dairy	15 (44.1)	1.0	0.52
	Both (dairy and beef)	21 (46.7)	1.4 (0.5–3.9)	
Type of husbandry	Intensive	31 (57.4)	7.9 (1.9–32.5)	0.004
	Semi-intensive	5 (20.0)	1.0	
Manure use	Use without biogas	30 (41.7)	1.0	0.06
	After biogas	6 (85.7)	10.2 (0.9–105.6)	
Silage feeding	Yes	4 (80.0)	5.2 (0.5–51.7)	0.16
	No	32 (43.2)	1.0	
New animal incursion	No	17 (38.6)	1.0	0.08
	Yes	19 (55.9)	2.4 (0.8–6.7)	
Vet service provider	Vet	14 (63.6)	2.7 (0.9–7.8)	0.07
	Para Vet/ Lay	22 (38.6)	1.0	
Other animals in herd	Yes	11 (61.1)	1.9 (0.6–6.0)	0.27
	No	23 (41.1)	1.00	
Bio-security status	Good	9 (60.0)	3.0 (0.6–14.5)	0.36
	Moderate	22 (44.0)	1.5 (0.4–5.4)	
	Poor	5 (35.7)	1.0	-
bTB history in the farm	No	29 (41.4)	1.0	
	Yes	7 (77.8)	5.9 (1.0–33.6)	0.04

**Table 5:** Univariable logistic regression analysis of animal level risk factors for bTB (N = 1865).

Risk factor	Category	TST positive (n)(%)	Odds ratio (95% CI)	P Value
Sex	Male	16 (5.2)	1.0	0.006
	Female	194(12.4)	2.4 (1.3–4.5)	-
Age	≤1 year	11 (4.6)	1.0	<0.001
	1–3 years	43 (6.0)	1.3 (0.6–2.7)	-
	3–6 years	116 (18.2)	2.81.4–5.8)	
	> 6 years	40 (14.7)	3.3 (1.5–7.0)	
Animal source	Farm	111 (10.4)	1.0	
	Bought	99 (12.5)	1.23 (0.92, 1.64)	0.156
Breed	Local	4 (2.9)	1.0	0.59
	Friesian cross	191 (12.5)	2.2 (0.696.83)	
	Shahiwal/ Sindhi cross	10 (7.4)	1.9 (0.41–8.21)	
	Other cross (Jersey/Brahma)	5 (8.5)	1.8 (0.55–6.9)	
Pregnancy status	No	89 (7.7)	1.0	>0.001
	Yes	121 (17.1)	2.0 (1.4, 2.8)	
Parity	0–2	93 (7.5)	1.0	<0.001
	2–5	104 (19.5)	1.9 (1.4–2.8)	
	>5	13 (14.0)	2.2 (1.0–4.4)	
BCS	Poor (BCS: 0–3)	5 (10.6)	0.8 (0.3–2.4)	0.84
	Good (BCS:>6)	149 (12.0)	1.1 (0.7–1.6)	
	Medium (BCS: 4–6)	56 (9.7)	1.0	

Compared with a herd size of 1–10, the odds of bTB were 22.8 (95% CI: 5.2–100.9) and 45.6 times (95% CI: 5.0–417.7) greater in herd sizes of >20–50 and > 50, respectively. The odds of bTB were 2.2 (95% CI: 1.0–4.5) and 2.5 times (95% CI: 1.1–5.4) higher in cattle aged >3–6 years and >6 years compared to cattle aged ≤1 year. Pregnancy increased the odds of bTB infection by 1.7 times (95% CI: 1.2–2.4) compared to non-pregnant cattle (Table 6). No confounding variable was found. All two-way interactions of the variables retained in the final mixed-effect model were non-significant. The intraclass correlation coefficient (ICC) was 39.0% (95% CI: 21.4–53.5).

**Table 6:** Factors retained in the final multivariable mixed effect logistic regression model of risk of bovine tuberculosis at animal level in Bangladesh (N = 1865 cattle).

Risk factor	Category	Estimate	SE	Odds ratio (95% CI)	P-value
Pregnancy status	No	Reference	-	1.0	-
	Yes	0.62	0.19	1.7 (1.2–2.4)	0.004
Age (Years)	Up to 1	Reference	-	1.0	-
	>1–3	0.09	0.38	1.1 (0.52–2.31)	0.81
	>3–6	0.77	0.37	2.2 (1.0–4.5)	0.04
	>6	0.90	0.40	2.5 (1.1–5.4)	0.03

## Discussion

In this study, we estimated herd and cattle level bTB prevalence in five dairy-intensive districts in Bangladesh and identified risk factors for bTB in cattle. A substantial proportion of cattle and herds tested positive, with herd size, age of individuals, and pregnancy status significantly associated with bTB reactor status in cattle in these selected districts in Bangladesh. The study further suggests that frequent screening of bTB of larger herds and especially targeting older and pregnant cattle could reduce within herd transmission and minimize the risk of zoonotic transmission of tuberculosis in Bangladesh context.

As a whole, herd level prevalence was found to be 45.6% (95% CI = 34.3–57.2%). No previous published report on herd level bTB in Bangladesh is available to compare to this estimate. In addition, 31% (11/36) of the positive herds had a within-herd bTB prevalence of ≥25%. Of note was the observation that of the 11 high-prevalence herds, 7 were located in Dhaka city. With an average density of 44,500 people per square kilometer, Dhaka ranks amongst the most densely populated cities in the world. While in general, most people within urban environments in Dhaka boil milk prior to consumption, individuals living in close proximity to bTB positive animals may be at increased risk for acquiring zoonotic TB infection via aerosol or direct transmission [34]. The herd level prevalence we observed was consistent with 44%; [35] and 52.2% [36] in recent studies in Ethiopia, but higher than other reports of bTB prevalence of 15–22.4% [37–39] in Ethiopia and India, and lower than that (91.7%) of [40] in Ethiopia and 8% in Northern Ireland [41].

We identified herd size as a potential risk factor for likelihood of bTB test-positivity at farm level. Size of herds has been reported as a risk factor for bTB [42–49], however in our study another explanation might be the study design in which more cattle were tested in larger herds, which increases the herd-level sensitivity of the SICTT in larger herds. More than two-thirds of the tested farms (68.4%) practiced intensive farming, of which most were larger herds (>10 cattle). Therefore, the chances of within-herd transmission of bTB in these herds is very high and can result in a very high within-herd prevalence [7]. This is demonstrated in two older (>10 year) cattle farms in Dhaka city corporation area, in which approximately 45% or 46% of the cattle were found to be positive for bTB. We found a high herd intraclass correlation coefficient (39%), suggesting strong variability of bTB positivity between herds but weak variability of bTB positivity among individual animals within a herd i.e. there was a significant clustering of bTB positive cases within a herd.

As a whole, animal level prevalence was found to be 11.3% (95% CI = 9.9–12.8%). This is substantially higher than reported previously studies that suggested between 2–7.8% reactor animals from different geographical locations and in different cattle breeds of Bangladesh [10, 12, 13, 50]. However, our result is much lower than the finding (of 27.7% bTB prevalence) of Islam et al. (2007) in breeding bulls in a smaller study (n = 127) [11]. The estimated prevalence of bTB in these selected districts is also higher than that reported in India (7.3%; 95% CI: 5.6–9.5) derived from a pooled prevalence estimate of bTB, which is based on a random-effects (RE) meta-regression model [51].

At animal level, age and pregnancy were found to be potential risk factors for bTB in Bangladesh. Two age group of cattle >3–6 and > 6 years were found to be at higher risk of bTB. Our findings are consistent with previous studies [45, 49, 52, 53]. The risk associated with increasing age likely reflects longer exposure time to the bTB infected cattle [5, 54].

Pregnancy status was found to be an important risk factor for bTB which corroborates findings of other authors [55, 56]. Pregnancy related immunosuppression may be responsible for acquiring bTB infection [57].

In this study, of 1865 tested animals, we observed dual skin responses in 303 cattle due to bTB and *Mycobacterium avium* subsp. *paratuberculosis* (MAP) or environmental mycobacterium infection, of which only 22.8% (n = 69) were found to be bTB positive (Fig 2 and S3 Table). It is pertinent to note that bTB skin test results may be confounded by either MAP coinfection or exposure to environmental mycobacteria [58–60] as immune responses to MAP/ environmental mycobacteria may mask bTB positivity status for a certain period of time when using the comparative skin test [61, 62]. The observation of positive skin responses at the avian site in 303 animals suspected to be caused by either MAP or environmental mycobacteria indicates that co-infection and/or co-exposure do occur. Therefore, how, when and for how long the ability to detect bTB using the SICTT is affected, needs further exploration.

To minimize the burden of further exposures mandatory practice of “test and slaughter policy” of test-positive farmed cattle which is an essential part of an annual screening program in high-income countries based on the identification of positive reactors and subsequent elimination of those reactors from the infected herd is currently absent in Bangladesh. However, the Government is implementing bTB screening activities—mostly in public farms, with little coverage at the private farm level.

Finally, we found that bTB is prevalent in cross bred cattle in periurban and urban areas of Bangladesh. In view of the financial losses caused by bTB and in addition to its public health risk, further efforts should be made to implement a science based disease control strategy. Test and slaughter has been presented as the best strategy to eliminate bTB in high income countries around the world [63]. However, due to socio-economic reasons such a strategy is not easily implementable in LMICs such as Bangladesh. Therefore, a wide variety of different options, including test and segregate or vaccination will have to be addressed and integrated into a fit for purpose strategy which can be implemented in Bangladesh.

A test and segregate strategy in the initial stages, and then move to test-and-slaughter methods in the final stage [1] after arrangement of adequate resources may represent a potential option for bTB control in Bangladesh. However, a critical need for compensation to minimize financial losses to cattle farmers may make this unfeasible as well. Awareness creation and motivation of cattle owners through participatory training in the critical areas of maintaining farm biosecurity measures, routine screening, and movement restriction and removal of infected herds are required for success of a control program [64]. Efforts for the development of BCG and other vaccines for control of bTB in cattle have recently shown considerable promise [65]. Given the lack of an established control program in Bangladesh, vaccination and similar approaches may be useful to consider implementing in Bangladesh through involvement of multisectoral collaboration among the veterinary and public health sectors to control the tuberculosis in source animal and subsequent transmission in humans is demanding [66, 67]. However, regardless of which control options are pursued, active surveillance together with on-farm visits, meat inspection in slaughterhouses, enhanced capabilities for on-field and laboratory diagnosis are critically needed to better inform the risks and consequences of un-controlled spread of bTB in Bangladesh to relevant stakeholders (animal health, human health, dairy industry, milk processors, policy planner, government authority, NGOs) so as to provide critical missing information to encourage implementation of bTB control together with that of other neglected zoonoses using a One Health approach [68, 69].

The primary limitations of this study was that this is a cross-sectional survey and only point prevalence estimates were obtained. Further, it was not possible to include all dairy intensive zones of Bangladesh to estimate true bTB burden and risk factors for the entire country. However, future studies of representative samples of herds and animals from other dairy intensive zones in Bangladesh are planned and recommended.

## Conclusions

This survey suggests a substantially high prevalence of bTB at the herd and animal levels in selected dairy intensive regions of Bangladesh and suggest an urgent need for the development of a comprehensive national strategy for control of bTB in high risk groups of cattle to minimize the risk of transmission from animals to humans and between animals.

## Supporting information

S1 Table Operational definitions and classification of herd level risk factors of bTB into levels. The weighting was completed in relation to bovine tuberculosis (bTB) positivity status.
(DOCX)

S2 Table Operational definitions and classification of animal level risk factors of bTB into levels. The weighting was completed in relation to bovine tuberculosis (bTB) positivity status of individual animal level.
(DOCX)

S3 Table Skin responses (N = 303) in mixed infections (bovine tuberculosis and paratuberculosis or environmental mycobacterium) that included tuberculin skin test positive (n = 69), negative (n = 188) and inclusive (n = 46) animal through measuring differences of skin thickness before and 72 h after tuberculin administration. (DOCX)

S1 Questionnaire Survey interview questionnaire for possible risk factors of bTB responsible for herd infection in urban and periurban areas of some selected districts of Bangladesh. (DOCX)

S2 Questionnaire Survey interview questionnaire for possible risk factors of bTB responsible for herd infection in urban and periurban areas of some selected districts of Bangladesh (Bengali version). (PDF)

S3 Questionnaire Survey interview questionnaire for possible risk factors of bTB responsible for animal level infection in urban and periurban areas of some selected district of Bangladesh. (DOCX)

S4 Questionnaire Survey interview for possible risk factors of bTB responsible for animal level infection in urban and periurban areas of some selected district of Bangladesh (Bengali version). (PDF)

S1 File OpenBUGS code to estimate animal level true prevalence of bTB. (TXT)
