# Correction: Bovine tuberculosis prevalence and risk factors in selected districts of Bangladesh

**DOI:** 10.1371/journal.pone.0256042

**Published:** 2021-08-05

**Authors:** SK Shaheenur Islam, Tanzida Begum Rumi, S. M. Lutful Kabir, Adri G. M. van der Zanden, Vivek Kapur, A. K. M. Anisur Rahman, Michael P. Ward, Douwe Bakker, Allen G. Ross, Zeaur Rahim

The first author’s name appears incorrectly. The correct name is: SK Shaheenur Islam.

The correct author contributions are:

Data curation: SK Shaheenur Islam, Tanzida Begum Rumi

Methodology: SK Shaheenur Islam, Tanzida Begum Rumi, Zeaur Rahim

Software: SK Shaheenur Islam, A. K. M. Anisur Rahman.

Writing–original draft: SK Shaheenur Islam, Tanzida Begum Rumi, A. K. M. Anisur

Rahman.

The correct citation is: Islam SS, Rumi TB, Kabir SML, van der Zanden AGM, Kapur V, Rahman AKMA, et al. (2020) Bovine tuberculosis prevalence and risk factors in selected districts of Bangladesh. PLoS ONE 15(11): e0241717. https://doi.org/10.1371/journal.pone.0241717
