# Supplementary material for: Bovine tuberculosis prevalence and risk factors in selected districts of Bangladesh
Source: PLoS One. 2020 Nov 10;15(11):e0241717. doi: 10.1371/journal.pone.0241717 (PMC7654795; doi:10.1371/journal.pone.0241717)
Supplement: S1 Table — The weighting was completed in relation to bovine tuberculosis (bTB) positivity status. (DOCX) [file pone.0241717.s001.docx]

**S1 Table.** Operational definitions and classification of herd level risk factors of bTB into levels. The weighting was completed in relation to bovine tuberculosis (bTB) positivity status.

|  | Study sites was defined as the districts included under this study: Dhaka, Mymensingh, Munshigang, Gazipur and Tangail districts |
| --- | --- |
|  | Age of the farms defined as number of years the farmer was rearing cattle. It was classified in three categories, 1-5 years, 5-10 years, and >10 years at cattle farming |
|  | Type of farms defined as the type of cattle was kept in the farms, tho categories, dairy: milk production, and dairy and beef (both): the farms kept both dairy and beef cattle for milk and meat production |
|  | The tentative bTB history of bTB referred as the previous status of a farm with bTB positivity and had three classes such as yes, no and do not know |
|  | Farm size was defined as the number of cattle being kept in a farm. Considering the farming practices of these districts farm size was classified into four (4) levels. Small: when the number of cattle between1-10; semi medium: between 10 and 20; medium: between 20 to 50; and large when>50 cattle. |
|  | Husbandry type defined three categories, intensive: cattle were kept in the farm for full time, semi-intensive: animal kept in both farm and outside (grazing), extensive: solely grazing in the field and kept in the farm during night. |
|  | Manure use purpose: two categories, directly use after dumping and store in open place and use in the agricultural/fish field as fertilizer, use after treatment in biogas plant: manure used as slurry after treatment in the agriculture field/vegetable gardening. |
|  | Silage feeding defined as use of processed grass as alternative source of cattle feed, two types, yes and no. |
|  | New animal inclusion defined as purchased new animal in the farm during the last 2 to 3 years from the study period, two categories, yes and no. If yes, then 2 categories, i.e. single and multiple sources. |
|  | Veterinary health care provider defined as who provide veterinary health care facilities during sickness of animal, vaccination, deworming along with castration etc, in two classes, are paraprofessional/paravet/lay/farmer himself/herself and vet. |
|  | Other animal keeping in the herd defined as other species like sheep, goat or poultry (mixed farming) kept within cattle in the farm. |
|  | Biosecurity type refers to the management practices that reduce the likelihood of bTB transmission to the herd. Neglect of such practices was hypothesized to increase the chances of bTB infection. To evaluate these practices on-farm we considered some indicator variables such as disposal of dead animals, cleaning and sanitation practices (of farm and premises, drainage system), distance to public habitat, entrance of dogs and other animals, presence of enclosures, and hygienic disposal of manure. Based on these indicators we categorized our studied farms into the three classes of poor; absence/no compliance with the above indicators, moderate: a minimum level of compliance with the above indicators, high: fully consistent with the above indicators |
