# Supplementary material for: Bovine tuberculosis prevalence and risk factors in selected districts of Bangladesh
Source: PLoS One. 2020 Nov 10;15(11):e0241717. doi: 10.1371/journal.pone.0241717 (PMC7654795; doi:10.1371/journal.pone.0241717)
Supplement: S2 Table — The weighting was completed in relation to bovine tuberculosis (bTB) positivity status of individual animal level. (DOCX) [file pone.0241717.s002.docx]

**S2 Table:** Operational definitions and classification of animal level risk factors of bTB into levels. The weighting was completed in relation to bovine tuberculosis (bTB) positivity status of individual animal level.

|  | Sex of the animal into two classes, either male or female |
| --- | --- |
|  | Age defined as how old the animal was during conducting SICTT |
|  | Source of animal defined as how the animal was included in the herd, two classes: ether born or bought |
|  | Breed: four classes: Frisian cross, Shahiwal/Sindhi cross, Other (Brahma/Jersey) cross and local/indigenous breed |
|  | Weight: Body weight (Kg) of the individual animal during SICTT in five categories, 1-100 Kg, 100-200 Kg, 200-400 Kg, 400-500 Kg, and >500 Kg |
|  | Milking status defined as female animal as milch cow, two categories: yes, no |
|  | Stage of lactation defined as, at what stages the milch cow was, classified at initial, mid, last |
|  | Pregnancy status defined as status of pregnancy in a cow, into two categories, yes, no |
|  | Parity defined as how many calves were gave birth, classified as 1-2 calving , 2-5 calving and >5 calving |
|  | Body condition score: Body condition scoring was categorized into three, viz. poor, medium and good BCS. A poor condition (BCS: 0-3) animal an angular and bony shape with no or negligible amount of fat over the backbone, ribs, pins and hooks. Moreover, there is no noticeable fat around the tail, head or even in the brisket. However, medium condition (BCS: 4-6) cattle have moderately good appearance, evident hips, though there is a small part of fat over the hooks, pins and the backbone is no longer detectable. Good condition cattle (BCS > 6) are fleshy and the ribs are no longer observable. There is also fat everywhere even in tail head and in the brisket |
