# Supplementary material for: Bovine tuberculosis prevalence and risk factors in selected districts of Bangladesh
Source: PLoS One. 2020 Nov 10;15(11):e0241717. doi: 10.1371/journal.pone.0241717 (PMC7654795; doi:10.1371/journal.pone.0241717)
Supplement: S3 Table — (DOCX) [file pone.0241717.s003.docx]

**S3 Table**. Skin responses (N=303) in mixed infections (bovine tuberculosis and paratuberculosis or environmental mycobacterium) that included tuberculin skin test positive (n=69), negative (n=188) and inclusive (n=46) animal through measuring differences of skin thickness before and 72 h after tuberculin administration.

| Animal no. | B1 | A1 | B2 | A2 | ΔB | ΔA | ΔB-ΔA | Interpretation at SICTT |
| --- | --- | --- | --- | --- | --- | --- | --- | --- |
| 1 | 7 | 6.5 | 7.5 | 9.5 | 0.5 | 3 | -2.5 | Negative |
| 2 | 7.5 | 7 | 8 | 8 | 0.5 | 1 | -0.5 | Negative |
| 3 | 5 | 4.5 | 6 | 8.5 | 1 | 4 | -3 | Negative |
| 4 | 9 | 8.5 | 9.5 | 10 | 0.5 | 1.5 | -1 | Negative |
| 5 | 6 | 6 | 13 | 9 | 7 | 3 | 4 | Inconclusive |
| 6 | 7 | 7 | 7.5 | 10 | 0.5 | 3 | -2.5 | Negative |
| 7 | 7 | 6 | 9.5 | 8 | 2.5 | 2 | 0.5 | Negative |
| 8 | 5 | 5 | 8.5 | 5.5 | 3.5 | 0.5 | 3 | Inconclusive |
| 9 | 6 | 5 | 7 | 6.5 | 1 | 1.5 | -0.5 | Negative |
| 10 | 7.5 | 7.5 | 13.5 | 11 | 6 | 3.5 | 2.5 | Inconclusive |
| 11 | 8 | 8 | 8.5 | 10 | 0.5 | 2 | -1.5 | Negative |
| 12 | 6 | 6 | 9.5 | 7 | 3.5 | 1 | 2.5 | Inconclusive |
| 13 | 7.5 | 8 | 8 | 11.5 | 0.5 | 3.5 | -3 | Negative |
| 14 | 4 | 4 | 6 | 5 | 2 | 1 | 1 | Negative |
| 15 | 10.5 | 8.5 | 19 | 9 | 8.5 | 0.5 | 8 | Positive |
| 16 | 7.5 | 7 | 12 | 7.5 | 4.5 | 0.5 | 4 | Inconclusive |
| 17 | 9 | 9 | 12 | 10 | 3 | 1 | 2 | Inconclusive |
| 18 | 8.5 | 7.5 | 15 | 8 | 6.5 | 0.5 | 6 | Positive |
| 19 | 7 | 8 | 18 | 10 | 11 | 2 | 9 | Positive |
| 20 | 10 | 7.5 | 18 | 8 | 8 | 0.5 | 7.5 | Positive |
| 21 | 6 | 6 | 22 | 8 | 16 | 2 | 14 | Positive |
| 22 | 5.5 | 5 | 9 | 9 | 3.5 | 4 | -0.5 | Negative |
| 23 | 7 | 6 | 11 | 8.5 | 4 | 2.5 | 1.5 | Negative |
| 24 | 12.5 | 11.5 | 18 | 13 | 5.5 | 1.5 | 4 | Inconclusive |
| 25 | 13.5 | 12 | 16 | 12.5 | 2.5 | 0.5 | 2 | Inconclusive |
| 26 | 6 | 3.5 | 23 | 4 | 17 | 0.5 | 16.5 | Positive |
| 27 | 5.5 | 4.5 | 22 | 5.5 | 16.5 | 1 | 15.5 | Positive |
| 28 | 16 | 12.5 | 23 | 17 | 7 | 4.5 | 2.5 | Inconclusive |
| 29 | 16 | 11 | 18 | 12.5 | 2 | 1.5 | 0.5 | Negative |
| 30 | 8.5 | 7.5 | 10 | 13.5 | 1.5 | 6 | -4.5 | Negative |
| 31 | 9.5 | 6.5 | 19 | 7 | 9.5 | 0.5 | 9 | Positive |
| 32 | 7 | 7 | 10 | 12 | 3 | 5 | -2 | Negative |
| 33 | 13.5 | 11 | 15 | 14 | 1.5 | 3 | -1.5 | Negative |
| 34 | 8 | 5 | 14 | 10 | 6 | 5 | 1 | Negative |
| 35 | 9 | 7.5 | 15 | 8 | 6 | 0.5 | 5.5 | Positive |
| 36 | 7.5 | 7 | 13.5 | 7.5 | 6 | 0.5 | 5.5 | Positive |
| 37 | 9 | 7 | 11.5 | 9 | 2.5 | 2 | 0.5 | Negative |
| 38 | 8.5 | 8 | 10 | 11 | 1.5 | 3 | -1.5 | Negative |
| 39 | 5.5 | 5.5 | 6.5 | 7 | 1 | 1.5 | -0.5 | Negative |
| 40 | 8 | 7 | 12 | 8 | 4 | 1 | 3 | Inconclusive |
| 41 | 8.5 | 7.5 | 11.5 | 8 | 3 | 0.5 | 2.5 | Inconclusive |
| 42 | 7.5 | 6 | 9 | 9 | 1.5 | 3 | -1.5 | Negative |
| 43 | 8 | 6.5 | 11 | 7 | 3 | 0.5 | 2.5 | Inconclusive |
| 44 | 7 | 7 | 18.5 | 8 | 11.5 | 1 | 10.5 | Positive |
| 45 | 8.5 | 10.5 | 11.5 | 11 | 3 | 0.5 | 2.5 | Inconclusive |
| 46 | 6 | 5.5 | 12 | 6 | 6 | 0.5 | 5.5 | Positive |
| 47 | 9 | 6 | 13 | 7.5 | 4 | 1.5 | 2.5 | Inconclusive |
| 48 | 8 | 7 | 12.5 | 7.5 | 4.5 | 0.5 | 4 | Inconclusive |
| 49 | 5.5 | 5.5 | 9 | 6 | 3.5 | 0.5 | 3 | Inconclusive |
| 50 | 8 | 7 | 10 | 8 | 2 | 1 | 1 | Negative |
| 51 | 10 | 9 | 11 | 12 | 1 | 3 | -2 | Negative |
| 52 | 5.5 | 5.5 | 6.5 | 7.5 | 1 | 2 | -1 | Negative |
| 53 | 5 | 5.5 | 9 | 10 | 4 | 4.5 | -0.5 | Negative |
| 54 | 7.5 | 7.5 | 10 | 10 | 2.5 | 2.5 | 0 | Negative |
| 55 | 10 | 9 | 13 | 13 | 3 | 4 | -1 | Negative |
| 56 | 5.5 | 5.5 | 6.5 | 7 | 1 | 1.5 | -0.5 | Negative |
| 57 | 8.5 | 8.5 | 12 | 12 | 3.5 | 3.5 | 0 | Negative |
| 58 | 7 | 6 | 7.5 | 7.5 | 0.5 | 1.5 | -1 | Negative |
| 59 | 5 | 5 | 6 | 7.5 | 1 | 2.5 | -1.5 | Negative |
| 60 | 4 | 4 | 17 | 6 | 13 | 2 | 11 | Positive |
| 61 | 6 | 5.5 | 17 | 6.5 | 11 | 1 | 10 | Positive |
| 62 | 6 | 6 | 21 | 12 | 15 | 6 | 9 | Positive |
| 63 | 6 | 5.5 | 13.5 | 6.5 | 7.5 | 1 | 6.5 | Positive |
| 64 | 5 | 5 | 14.5 | 9.5 | 9.5 | 4.5 | 5 | Positive |
| 65 | 9 | 7.5 | 11 | 11 | 2 | 3.5 | -1.5 | Negative |
| 66 | 5.5 | 5 | 18 | 9 | 12.5 | 4 | 8.5 | Positive |
| 67 | 5 | 5 | 17 | 10 | 12 | 5 | 7 | Positive |
| 68 | 5.5 | 5.5 | 14 | 8 | 8.5 | 2.5 | 6 | Positive |
| 69 | 6 | 6 | 31 | 11 | 25 | 5 | 20 | Positive |
| 70 | 6 | 6 | 12 | 6.5 | 6 | 0.5 | 5.5 | Positive |
| 71 | 6 | 5.5 | 9 | 8 | 3 | 2.5 | 0.5 | Negative |
| 72 | 3.5 | 3.5 | 5 | 7 | 1.5 | 3.5 | -2 | Negative |
| 73 | 6 | 6 | 17 | 10 | 11 | 4 | 7 | Positive |
| 74 | 4 | 4 | 6 | 5 | 2 | 1 | 1 | Negative |
| 75 | 5.5 | 5.5 | 10 | 6 | 4.5 | 0.5 | 4 | Inconclusive |
| 76 | 6 | 6 | 21 | 12.5 | 15 | 6.5 | 8.5 | Positive |
| 77 | 7 | 6 | 10.5 | 9 | 3.5 | 3 | 0.5 | Negative |
| 78 | 7 | 6 | 14 | 7 | 7 | 1 | 6 | Positive |
| 79 | 5.5 | 5.5 | 7 | 8 | 1.5 | 2.5 | -1 | Negative |
| 80 | 6.5 | 6 | 7 | 7 | 0.5 | 1 | -0.5 | Negative |
| 81 | 8 | 7.5 | 9 | 8 | 1 | 0.5 | 0.5 | Negative |
| 82 | 9 | 7 | 12 | 8.5 | 3 | 1.5 | 1.5 | Negative |
| 83 | 6 | 5.5 | 7.5 | 6.5 | 1.5 | 1 | 0.5 | Negative |
| 84 | 5 | 5 | 14 | 9 | 9 | 4 | 5 | Positive |
| 85 | 6 | 6 | 13 | 8 | 7 | 2 | 5 | Positive |
| 86 | 7.5 | 5.5 | 17 | 12 | 9.5 | 6.5 | 3 | Inconclusive |
| 87 | 5 | 5 | 15 | 8 | 10 | 3 | 7 | Positive |
| 88 | 5.5 | 5.5 | 11.5 | 7 | 6 | 1.5 | 4.5 | Positive |
| 89 | 8 | 8 | 15 | 10 | 7 | 2 | 5 | Positive |
| 90 | 7 | 6.5 | 20 | 7.5 | 13 | 1 | 12 | Positive |
| 91 | 8 | 6.5 | 13 | 9 | 5 | 2.5 | 2.5 | Inconclusive |
| 92 | 8 | 7 | 10 | 7.5 | 2 | 0.5 | 1.5 | Negative |
| 93 | 7.5 | 7.5 | 14 | 10 | 6.5 | 2.5 | 4 | Inconclusive |
| 94 | 8 | 7 | 18 | 8 | 10 | 1 | 9 | Positive |
| 95 | 8.5 | 7.5 | 10 | 9.5 | 1.5 | 2 | -0.5 | Negative |
| 96 | 10.5 | 9.5 | 11 | 12 | 0.5 | 2.5 | -2 | Negative |
| 97 | 9.5 | 8 | 13.5 | 10.5 | 4 | 2.5 | 1.5 | Negative |
| 98 | 6.5 | 5.5 | 7.5 | 8 | 1 | 2.5 | -1.5 | Negative |
| 99 | 5 | 5.5 | 8 | 8 | 3 | 2.5 | 0.5 | Negative |
| 100 | 8 | 7 | 17 | 11 | 9 | 4 | 5 | Positive |
| 101 | 6 | 5 | 11 | 9 | 5 | 4 | 1 | Negative |
| 102 | 7.5 | 7.5 | 9 | 8 | 1.5 | 0.5 | 1 | Negative |
| 103 | 6.5 | 6 | 12 | 8 | 5.5 | 2 | 3.5 | Inconclusive |
| 104 | 8 | 7 | 9.5 | 9.5 | 1.5 | 2.5 | -1 | Negative |
| 105 | 10.5 | 9 | 17 | 15 | 6.5 | 6 | 0.5 | Negative |
| 106 | 7.5 | 6.5 | 9 | 9 | 1.5 | 2.5 | -1 | Negative |
| 107 | 7 | 7 | 7.5 | 9 | 0.5 | 2 | -1.5 | Negative |
| 108 | 9 | 8 | 10.5 | 10 | 1.5 | 2 | -0.5 | Negative |
| 109 | 9 | 9 | 24 | 11 | 15 | 2 | 13 | Positive |
| 110 | 9 | 8 | 11 | 11 | 2 | 3 | -1 | Negative |
| 111 | 8 | 6 | 11 | 12 | 3 | 6 | -3 | Negative |
| 112 | 10.5 | 10 | 11 | 11 | 0.5 | 1 | -0.5 | Negative |
| 113 | 9 | 8 | 11 | 10 | 2 | 2 | 0 | Negative |
| 114 | 7 | 6 | 11 | 10 | 4 | 4 | 0 | Negative |
| 115 | 6 | 5 | 8 | 7 | 2 | 2 | 0 | Negative |
| 116 | 5.5 | 6 | 8 | 9 | 2.5 | 3 | -0.5 | Negative |
| 117 | 4.5 | 4 | 10 | 8 | 5.5 | 4 | 1.5 | Negative |
| 118 | 8.5 | 8.5 | 12.5 | 11 | 4 | 2.5 | 1.5 | Negative |
| 119 | 10 | 8.5 | 14.5 | 12 | 4.5 | 3.5 | 1 | Negative |
| 120 | 8 | 7 | 12 | 10.5 | 4 | 3.5 | 0.5 | Negative |
| 121 | 7 | 5.5 | 8 | 8 | 1 | 2.5 | -1.5 | Negative |
| 122 | 5 | 5 | 11 | 11 | 6 | 6 | 0 | Negative |
| 123 | 10 | 7 | 13 | 10 | 3 | 3 | 0 | Negative |
| 124 | 8 | 8 | 12 | 12.5 | 4 | 4.5 | -0.5 | Negative |
| 125 | 3.5 | 3 | 4 | 5.5 | 0.5 | 2.5 | -2 | Negative |
| 126 | 12 | 10 | 14 | 10.5 | 2 | 0.5 | 1.5 | Negative |
| 127 | 5.5 | 5.5 | 10 | 7 | 4.5 | 1.5 | 3 | Inconclusive |
| 128 | 5 | 5.5 | 11 | 7.5 | 6 | 2 | 4 | Inconclusive |
| 129 | 7.5 | 7 | 17 | 10 | 9.5 | 3 | 6.5 | Positive |
| 130 | 6 | 6 | 17 | 9.5 | 11 | 3.5 | 7.5 | Positive |
| 131 | 7 | 5 | 20 | 7 | 13 | 2 | 11 | Positive |
| 132 | 5 | 4.5 | 19 | 6.5 | 14 | 2 | 12 | Positive |
| 133 | 6.5 | 6 | 14 | 6.5 | 7.5 | 0.5 | 7 | Positive |
| 134 | 5 | 5 | 11 | 6.5 | 6 | 1.5 | 4.5 | Positive |
| 135 | 7.5 | 7 | 14 | 10.5 | 6.5 | 3.5 | 3 | Inconclusive |
| 136 | 7.5 | 7 | 18 | 11 | 10.5 | 4 | 6.5 | Positive |
| 137 | 7 | 5 | 14 | 9 | 7 | 4 | 3 | Inconclusive |
| 138 | 6.5 | 5 | 17 | 7 | 10.5 | 2 | 8.5 | Positive |
| 139 | 6 | 5.5 | 18 | 7 | 12 | 1.5 | 10.5 | Positive |
| 140 | 8.5 | 7 | 15 | 9 | 6.5 | 2 | 4.5 | Positive |
| 141 | 6 | 5.5 | 16 | 9 | 10 | 3.5 | 6.5 | Positive |
| 142 | 8 | 7 | 21 | 10 | 13 | 3 | 10 | Positive |
| 143 | 8.5 | 8 | 15 | 10 | 6.5 | 2 | 4.5 | Positive |
| 144 | 7.5 | 7 | 11 | 9 | 3.5 | 2 | 1.5 | Negative |
| 145 | 7.5 | 7 | 20 | 10 | 12.5 | 3 | 9.5 | Positive |
| 146 | 8 | 7 | 17 | 10 | 9 | 3 | 6 | Positive |
| 147 | 8 | 8 | 16 | 10 | 8 | 2 | 6 | Positive |
| 148 | 9 | 7 | 32 | 13 | 23 | 6 | 17 | Positive |
| 149 | 7 | 7 | 10.5 | 10 | 3.5 | 3 | 0.5 | Negative |
| 150 | 8 | 6.5 | 21 | 13 | 13 | 6.5 | 6.5 | Positive |
| 151 | 6 | 5 | 9 | 6 | 3 | 1 | 2 | Inconclusive |
| 152 | 9.5 | 8 | 11 | 11 | 1.5 | 3 | -1.5 | Negative |
| 153 | 5 | 4 | 8 | 6 | 3 | 2 | 1 | Negative |
| 154 | 4.5 | 4 | 10 | 7 | 5.5 | 3 | 2.5 | Inconclusive |
| 155 | 6 | 6 | 11.5 | 7 | 5.5 | 1 | 4.5 | Positive |
| 156 | 6.5 | 6.5 | 7 | 7 | 0.5 | 0.5 | 0 | Negative |
| 157 | 8.5 | 7 | 9.5 | 10 | 1 | 3 | -2 | Negative |
| 158 | 8 | 6 | 9.5 | 8 | 1.5 | 2 | -0.5 | Negative |
| 159 | 15 | 12 | 18.5 | 15 | 3.5 | 3 | 0.5 | Negative |
| 160 | 5.5 | 5 | 7.5 | 9 | 2 | 4 | -2 | Negative |
| 161 | 8 | 6 | 11.5 | 9 | 3.5 | 3 | 0.5 | Negative |
| 162 | 5.5 | 5 | 6 | 6 | 0.5 | 1 | -0.5 | Negative |
| 163 | 4 | 3.5 | 7 | 6 | 3 | 2.5 | 0.5 | Negative |
| 164 | 5.5 | 6 | 9 | 8 | 3.5 | 2 | 1.5 | Negative |
| 165 | 9 | 8 | 15 | 11 | 6 | 3 | 3 | Inconclusive |
| 166 | 6 | 5 | 11 | 9 | 5 | 4 | 1 | Negative |
| 167 | 7 | 5.5 | 9.5 | 7 | 2.5 | 1.5 | 1 | Negative |
| 168 | 7 | 7 | 13 | 11 | 6 | 4 | 2 | Inconclusive |
| 169 | 7 | 6 | 15 | 8 | 8 | 2 | 6 | Positive |
| 170 | 8 | 6 | 11.5 | 8 | 3.5 | 2 | 1.5 | Negative |
| 171 | 8.5 | 8 | 11 | 9 | 2.5 | 1 | 1.5 | Negative |
| 172 | 7 | 5 | 11 | 8 | 4 | 3 | 1 | Negative |
| 173 | 7 | 6 | 15 | 10 | 8 | 4 | 4 | Inconclusive |
| 174 | 5 | 4 | 8 | 6 | 3 | 2 | 1 | Negative |
| 175 | 5 | 4 | 9 | 6 | 4 | 2 | 2 | Inconclusive |
| 176 | 5 | 5 | 8 | 6.5 | 3 | 1.5 | 1.5 | Negative |
| 177 | 6.5 | 5.5 | 9 | 8 | 2.5 | 2.5 | 0 | Negative |
| 178 | 5 | 4 | 6 | 6 | 1 | 2 | -1 | Negative |
| 179 | 8 | 6 | 10 | 11 | 2 | 5 | -3 | Negative |
| 180 | 3.5 | 3 | 12 | 6 | 8.5 | 3 | 5.5 | Positive |
| 181 | 8 | 6 | 12 | 7 | 4 | 1 | 3 | Inconclusive |
| 182 | 6.5 | 6 | 8 | 9 | 1.5 | 3 | -1.5 | Negative |
| 183 | 5 | 4 | 7 | 6 | 2 | 2 | 0 | Negative |
| 184 | 5 | 4 | 7.5 | 7 | 2.5 | 3 | -0.5 | Negative |
| 185 | 5.5 | 4 | 7 | 6 | 1.5 | 2 | -0.5 | Negative |
| 186 | 4 | 3.5 | 23 | 7 | 19 | 3.5 | 15.5 | Positive |
| 187 | 6.5 | 6 | 15 | 10 | 8.5 | 4 | 4.5 | Positive |
| 188 | 6 | 6 | 15 | 10 | 9 | 4 | 5 | Positive |
| 189 | 7 | 9 | 9 | 13 | 2 | 4 | -2 | Negative |
| 190 | 4 | 4 | 6 | 6.5 | 2 | 2.5 | -0.5 | Negative |
| 191 | 4 | 4 | 5 | 8 | 1 | 4 | -3 | Negative |
| 192 | 5.5 | 5 | 11 | 8 | 5.5 | 3 | 2.5 | Inconclusive |
| 193 | 6 | 5 | 10.5 | 7 | 4.5 | 2 | 2.5 | Inconclusive |
| 194 | 6 | 5 | 7.5 | 7.5 | 1.5 | 2.5 | -1 | Negative |
| 195 | 9 | 7 | 13 | 9 | 4 | 2 | 2 | Inconclusive |
| 196 | 4 | 4 | 5.5 | 7.5 | 1.5 | 3.5 | -2 | Negative |
| 197 | 6 | 7 | 7 | 9 | 1 | 2 | -1 | Negative |
| 198 | 5.5 | 4.5 | 7.5 | 7 | 2 | 2.5 | -0.5 | Negative |
| 199 | 6 | 5 | 6.5 | 10 | 0.5 | 5 | -4.5 | Negative |
| 200 | 8 | 7 | 10 | 8 | 2 | 1 | 1 | Negative |
| 201 | 4 | 3 | 6 | 6 | 2 | 3 | -1 | Negative |
| 202 | 4 | 3 | 5 | 4 | 1 | 1 | 0 | Negative |
| 203 | 6 | 5 | 7 | 10 | 1 | 5 | -4 | Negative |
| 204 | 6 | 4.5 | 9 | 5 | 3 | 0.5 | 2.5 | Inconclusive |
| 205 | 8.5 | 7 | 9 | 8 | 0.5 | 1 | -0.5 | Negative |
| 206 | 7 | 5 | 10.5 | 6 | 3.5 | 1 | 2.5 | Inconclusive |
| 207 | 9 | 7 | 12 | 10 | 3 | 3 | 0 | Negative |
| 208 | 4 | 4 | 5 | 7 | 1 | 3 | -2 | Negative |
| 209 | 7 | 5 | 8.5 | 7 | 1.5 | 2 | -0.5 | Negative |
| 210 | 4.5 | 4 | 5 | 5 | 0.5 | 1 | -0.5 | Negative |
| 211 | 13 | 11 | 14 | 12 | 1 | 1 | 0 | Negative |
| 212 | 10 | 8.5 | 11 | 9 | 1 | 0.5 | 0.5 | Negative |
| 213 | 11 | 8 | 12 | 10 | 1 | 2 | -1 | Negative |
| 214 | 8.5 | 5.5 | 9 | 7 | 0.5 | 1.5 | -1 | Negative |
| 215 | 10 | 8 | 12 | 10 | 2 | 2 | 0 | Negative |
| 216 | 5.5 | 5 | 6.5 | 7.5 | 1 | 2.5 | -1.5 | Negative |
| 217 | 5 | 4.5 | 8.5 | 6.5 | 3.5 | 2 | 1.5 | Negative |
| 218 | 5.5 | 5 | 12 | 7 | 6.5 | 2 | 4.5 | Positive |
| 219 | 5.5 | 5 | 9 | 6 | 3.5 | 1 | 2.5 | Inconclusive |
| 220 | 3.5 | 3.5 | 3.5 | 3.5 | 0.5 | 0.5 | 0 | Negative |
| 221 | 3 | 3 | 11.5 | 6 | 8.5 | 1 | 7.5 | Positive |
| 222 | 3 | 5 | 8 | 6 | 5 | 1 | 4 | Inconclusive |
| 223 | 7 | 5 | 9 | 10 | 2 | 5 | -3 | Negative |
| 224 | 5 | 5 | 7.5 | 7.5 | 2.5 | 2.5 | 0 | Negative |
| 225 | 6.5 | 6.5 | 7 | 8.5 | 0.5 | 2 | -1.5 | Negative |
| 226 | 10 | 7 | 11 | 8.5 | 1 | 1.5 | -0.5 | Negative |
| 227 | 4.5 | 4 | 7 | 8 | 2.5 | 4 | -1.5 | Negative |
| 228 | 9 | 8 | 11 | 10 | 2 | 2 | 0 | Negative |
| 229 | 5 | 5 | 6 | 9 | 1 | 4 | -3 | Negative |
| 230 | 5.5 | 5.5 | 6 | 7.5 | 0.5 | 2 | -1.5 | Negative |
| 231 | 5 | 5 | 8 | 8.5 | 3 | 3.5 | -0.5 | Negative |
| 232 | 3 | 3 | 4 | 5 | 1 | 2 | -1 | Negative |
| 233 | 9 | 7 | 19 | 9.5 | 10 | 2.5 | 7.5 | Positive |
| 234 | 10 | 8 | 15 | 10.5 | 5 | 2.5 | 2.5 | Negative |
| 235 | 7 | 6 | 8 | 9.5 | 1 | 3.5 | -2.5 | Negative |
| 236 | 10 | 8 | 11 | 11 | 1 | 3 | -2 | Negative |
| 237 | 13.5 | 10 | 15 | 11 | 1.5 | 1 | 0.5 | Negative |
| 238 | 8 | 6 | 8.5 | 7.5 | 0.5 | 1.5 | -1 | Negative |
| 239 | 6 | 6 | 12 | 7.5 | 6 | 1.5 | 4.5 | Positive |
| 240 | 5 | 5 | 12 | 7.5 | 7 | 2.5 | 4.5 | Positive |
| 241 | 5 | 5 | 12 | 6.5 | 7 | 1.5 | 5.5 | Positive |
| 242 | 7 | 7 | 9.5 | 12 | 2.5 | 5 | -2.5 | Negative |
| 243 | 7 | 6 | 9 | 9.5 | 2 | 3.5 | -1.5 | Negative |
| 244 | 7 | 6 | 12.5 | 7 | 5.5 | 1 | 4.5 | Positive |
| 245 | 7 | 7 | 15.5 | 11 | 8.5 | 4 | 4.5 | Positive |
| 246 | 6 | 6 | 10.5 | 8 | 4.5 | 2 | 2.5 | Inconclusive |
| 247 | 7.5 | 8.5 | 11 | 10 | 3.5 | 1.5 | 2 | Inconclusive |
| 248 | 9 | 8 | 16 | 9 | 7 | 1 | 6 | Positive |
| 249 | 7 | 6 | 11 | 7.5 | 4 | 1.5 | 2.5 | Inconclusive |
| 250 | 5.5 | 4.5 | 9 | 6.5 | 3.5 | 2 | 1.5 | Negative |
| 251 | 9 | 7 | 10 | 10 | 1 | 3 | -2 | Negative |
| 252 | 6 | 6 | 11.5 | 7 | 5.5 | 1 | 4.5 | Positive |
| 253 | 6 | 6 | 11 | 9 | 5 | 3 | 2 | Inconclusive |
| 254 | 4 | 6 | 7 | 10 | 3 | 4 | -1 | Negative |
| 255 | 7.5 | 7 | 12.5 | 10 | 5 | 3 | 2 | Inconclusive |
| 256 | 5 | 5 | 6 | 7 | 1 | 2 | -1 | Negative |
| 257 | 6 | 5.5 | 7 | 6 | 1 | 0.5 | 0.5 | Negative |
| 258 | 5 | 4 | 7 | 5 | 2 | 1 | 1 | Negative |
| 259 | 3 | 3 | 5 | 4 | 2 | 1 | 1 | Negative |
| 260 | 5 | 4 | 6 | 5 | 1 | 1 | 0 | Negative |
| 261 | 6 | 5 | 9 | 6 | 3 | 1 | 2 | Inconclusive |
| 262 | 4.5 | 4.5 | 5 | 5 | 0.5 | 0.5 | 0 | Negative |
| 263 | 3 | 3 | 4 | 4 | 1 | 1 | 0 | Negative |
| 264 | 8 | 8 | 10 | 12 | 2 | 4 | -2 | Negative |
| 265 | 4 | 3.5 | 5 | 4 | 1 | 0.5 | 0.5 | Negative |
| 266 | 4 | 4 | 5 | 5 | 1 | 1 | 0 | Negative |
| 267 | 4 | 4 | 5 | 6 | 1 | 2 | -1 | Negative |
| 268 | 9 | 7 | 11 | 8 | 2 | 1 | 1 | Negative |
| 269 | 3 | 3 | 3.5 | 3.5 | 0.5 | 0.5 | 0 | Negative |
| 270 | 3 | 4 | 4 | 9 | 1 | 5 | -4 | Negative |
| 271 | 3 | 3 | 4 | 4 | 1 | 1 | 0 | Negative |
| 272 | 8 | 7 | 9 | 9 | 1 | 2 | -1 | Negative |
| 273 | 4 | 4 | 5 | 5 | 1 | 1 | 0 | Negative |
| 274 | 5 | 4 | 6 | 4.5 | 1 | 0.5 | 0.5 | Negative |
| 275 | 4 | 3 | 5 | 5 | 1 | 2 | -1 | Negative |
| 276 | 5 | 4 | 6 | 6 | 1 | 2 | -1 | Negative |
| 277 | 3 | 3 | 3.5 | 6.5 | 0.5 | 3.5 | -3 | Negative |
| 278 | 3 | 3 | 4 | 4 | 1 | 1 | 0 | Negative |
| 279 | 3.5 | 3 | 5 | 6.5 | 1.5 | 3.5 | -2 | Negative |
| 280 | 3.5 | 4 | 6 | 4.5 | 2.5 | 0.5 | 2 | Inconclusive |
| 281 | 6 | 6 | 7.5 | 7 | 1.5 | 1 | 0.5 | Negative |
| 282 | 6 | 6 | 6.5 | 6.5 | 0.5 | 0.5 | 0 | Negative |
| 283 | 5 | 5 | 6.5 | 9 | 1.5 | 4 | -2.5 | Negative |
| 284 | 4 | 4 | 6 | 5 | 2 | 1 | 1 | Negative |
| 285 | 8 | 6 | 9 | 7 | 1 | 1 | 0 | Negative |
| 286 | 4.5 | 4.5 | 5 | 5 | 0.5 | 0.5 | 0 | Negative |
| 287 | 4.5 | 4 | 5 | 6 | 0.5 | 2 | -1.5 | Negative |
| 288 | 4 | 4 | 6 | 7 | 2 | 3 | -1 | Negative |
| 289 | 4.5 | 4.5 | 5 | 5 | 0.5 | 0.5 | 0 | Negative |
| 290 | 3.5 | 3.5 | 4 | 5 | 0.5 | 1.5 | -1 | Negative |
| 291 | 3 | 3 | 5 | 6 | 2 | 3 | -1 | Negative |
| 292 | 4.5 | 5 | 6.5 | 6.5 | 2 | 1.5 | 0.5 | Negative |
| 293 | 3.5 | 3.5 | 10 | 5 | 6.5 | 1.5 | 5 | Positive |
| 294 | 4 | 4 | 5 | 6 | 1 | 2 | -1 | Negative |
| 295 | 3.5 | 3 | 4 | 5 | 0.5 | 2 | -1.5 | Negative |
| 296 | 4 | 3.5 | 4.5 | 6 | 0.5 | 2.5 | -2 | Negative |
| 297 | 5 | 4 | 6.5 | 6 | 1.5 | 2 | -0.5 | Negative |
| 298 | 4 | 4 | 5 | 6 | 1 | 2 | -1 | Negative |
| 299 | 5 | 3.5 | 7 | 7 | 2 | 3.5 | -1.5 | Negative |
| 300 | 7 | 5 | 10 | 8 | 3 | 3 | 0 | Negative |
| 301 | 4 | 4 | 5 | 5 | 1 | 1 | 0 | Negative |
| 302 | 6 | 5 | 7 | 6 | 1 | 1 | 0 | Negative |
| 303 | 3 | 3 | 4 | 5 | 1 | 2 | -1 | Negative |

Note:

B1: Skin thickness (mm) in bovine site before tuberculin administration

A1: Skin thickness (mm) in avian site before tuberculin administration

B2: Skin thickness (mm) in bovine site after 72 h of tuberculin administration

A2: Skin thickness (mm) in avian site after 72 h of tuberculin administration

ΔB: Difference of skin thickness (mm) in bovine site after 72 h and before tuberculin administration

ΔA: Difference of skin thickness (mm) in avian site after 72 h and before tuberculin administration

ΔB-ΔA: if >4 mm is positive, 2-4 mm inconclusive, <2 mm Negative
