# Supplementary material for: Bovine tuberculosis prevalence and risk factors in selected districts of Bangladesh
Source: PLoS One. 2020 Nov 10;15(11):e0241717. doi: 10.1371/journal.pone.0241717 (PMC7654795; doi:10.1371/journal.pone.0241717)
Supplement: S1 Questionnaire — (DOCX) [file pone.0241717.s004.docx]

**S1 Questionnaire.** Survey interview questionnaire for possible risk factors of bTB responsible for herd infection in urban and periurban areas of some selected districts of Bangladesh.

|  |  |  |  |  |  | ID No: |  | |  |
| --- | --- | --- | --- | --- | --- | --- | --- | --- | --- |
| Date of Interview: | | ………/………/……….2019 | | |  |  |  |  |  |
| 1. GPS Coordinates: | | a) Northing | |  | b) Easting: | |  |  |  |
|  |  |  |  |  |  |  |  |  |  |
| 2. Name of the interviewee: | | |  | | Mobile number: | |  | | |
|  |  |  |  |  |  |  |  |  |  |
| 3. Name of the farm…………… | | | 3.1 | Date of establishment of the farm | | | ……/……../………… | |  |
|  |  |  |  |  |  |  |  |  |  |
| 4. Farm location: | |  |  |  |  |  |  |  |  |
|  | Village/road: |  | |  | Union/ Ward: |  |  |  |  |
|  |  |  |  |  |  |  |  |  |  |
|  | Upazila: |  | |  | District: |  |  |  |  |
|  |  |  |  |  |  |  |  |  |  |
| 5. Type of farm: | | Dairy |  |  | 6. History of TB of the farm/herd | | | |  |
|  |  |  |  |  |  |  |  |  |  |
|  |  | Fattening |  |  |  | Yes |  | Don’t Know | |
|  |  |  |  |  |  |  |  |  |  |
|  |  | Both |  |  |  | No |  |  |  |
|  |  |  |  |  |  |  |  |  |  |
| 7.Herd | 7.1 Herd Size | ………… | Cattle |  |  | 8. Type of husbandry? | | |  |
|  |  |  |  |  |  |  |  |  |  |
|  | 7.2 Beed |  |  |  |  | Intensive |  |  |  |
|  | Frisian Cross |  |  |  |  |  |  |  |  |
|  |  |  |  |  | Semi intensive | |  |  |  |
|  | Shahiwal Cross | |  |  |  |  |  |  |  |
|  |  |  |  |  |  | Extensive |  |  |  |
|  | Other Cross |  |  |  |  |  |  |  |  |
|  |  |  |  |  |  |  |  |  |  |
|  | Indigenous breed | |  |  |  |  |  |  |  |
| 9. Manure use method | | |  | 10. Feeding silage in intensive or semi-intensive system? | | | | | |
|  |  |  |  |  |  |  |  |  |  |
|  |  | Use directly | |  |  | Yes |  |  |  |
|  |  |  |  |  |  |  |  |  |  |
|  | Use after treatment | | |  |  | No |  |  |  |
|  |  |  |  |  |  |  |  |  |  |
| 11. New animal inclusion in the herd? | | | |  | 11.1 If Yes, how many sources of animal included? | | | | |
|  |  |  |  |  |  |  |  |  |  |
|  |  | Yes |  |  |  | Single |  |  |  |
|  |  |  |  |  |  |  |  |  |  |
|  |  | No |  |  | More than one | |  |  |  |
|  |  |  |  |  |  |  |  |  |  |
| 12. Veterinary healthcare provider | | | |  | 13 | Provision of mix (cattle/sheep/poultry) | | | |
|  |  |  |  |  |  |  |  |  |  |
|  | Paraprofessional or Lay | | |  |  | Yes |  |  |  |
|  |  |  |  |  |  |  |  |  |  |
|  | Veterinarian | | |  |  | No |  |  |  |
|  |  |  |  |  |  |  |  |  |  |
|  | Farmer himself/herself | | |  |  |  |  |  |  |
|  |  |  |  |  |  |  |  |  |  |
| 14. Biosecurity status? | | |  |  |  |  |  |  |  |
|  |  | Good |  |  |  |  |  |  |  |
|  |  |  |  |  |  |  |  |  |  |
|  |  | Bad |  |  |  |  |  |  |  |
|  |  |  |  |  |  |  |  |  |  |
|  |  | Medium |  |  |  |  |  |  |  |
|  |  |  |  |  |  |  |  |  |  |
