# Supplementary material for: Bovine tuberculosis prevalence and risk factors in selected districts of Bangladesh
Source: PLoS One. 2020 Nov 10;15(11):e0241717. doi: 10.1371/journal.pone.0241717 (PMC7654795; doi:10.1371/journal.pone.0241717)
Supplement: S2 Questionnaire — (PDF) [file pone.0241717.s005.pdf]

এস ৩ প্রশ্নাবলী। গবাদিপশুতে পাল/দল পর্যায়ে টিবি রোগ সংক্রমণ ঝুঁকি বিষয়ক সার্ভে প্রশ্নমালা

(সাক্ষাৎকার গ্রহণকারী কর্তৃক পূরনকৃত)

আইডি নং [ ]

সাক্ষাতকার গ্রহনের তারিখ: ..../........./.........২০...

১। জিপিএস এ ফার্ম এর অবস্থানঃ ক) নরথিং [ ] খ) ইসটিং [ ]

২। তথ্য প্রদানকারী ব্যক্তির নাম: ------------------- [ ] মোবাইল নং:

৩। খামারের নামঃ.................... ৩.১। খামার স্থাপনের তারিখ ....../......../............

৪। খামারের অবস্থানঃ

গ্রাম/ ওয়ার্ড [ ] ইউনিয়ন/ পৌরসভা [ ]

উপজেলা [ ] জেলা [ ]

৫। খামারের ধনর
- দুধ উৎপাদন [ ]
- মাংস উৎপাদন/ হৃষ্টপুষ্টকরণ [ ]
- উভয় [ ]

৬। গবাদিপশুতে টিবি আক্রান্ত হবার ইতিহাস আছে কি?
- [ ] হ্যাঁ
- [ ] না
- [ ] জানি না

৭। দল/পাল সংকান্ত তথ্যাদি

৭.১। পালের আকার ....টি গরু

৭.২। জাত
- ফ্রিজিয়ান ক্রস [ ]
- শাহীওয়াল ক্রস [ ]
- অন্যান্য ক্রস [ ]
- দেশী জাত [ ]

৮। লালন পালনের প্রকৃতি
- নিবিড় (গবাদিপশু কোন প্রকার বাহিরে চরানো হয় না) [ ]
- আধা নিবিড় (মাঝে মধ্যে চরিয়ে খাওয়ানো হয়) [ ]
- পুরোপুরি মাঠে চরিয়ে খাওয়ানো হয়। [ ]

৯। গোবর সার ব্যবহার পদ্ধতি
- কোন প্রকার ট্রিটমেন্ট ছাড়া সরাসরি ব্যবহার করা হয় [ ]
- বায়োগাস প্লান্টে ব্যবহারের পর স্লারি ব্যবহার করা হয় [ ]

১০। নিবিড় বা আধা নিবিড় পদ্ধতিতে সাইলেজ খাওয়ানো হয় কি?
- হ্যাঁ [ ]
- না [ ]

১১। পালে নতুন পশু আনা হয়েছে কি?
- হ্যাঁ [ ]
- না [ ]

১১.১। হ্যাঁ হলে উৎস
- এক [ ]
- একাধিক [ ]

১২। ভেটেরিনারি চিকিৎসা সেবা প্রদান কারী?

কোয়াক/ প্যারাপ্রফেশনাল	☐
ভেটেরিনারিয়ান	☐
খামারী নিজে	☐

১৩। খামারে অন্য প্রাণি (ছাগল/ভেড়া/ ইত্যাদি) রাখা হয়কি ?

হ্যাঁ	☐
হ্যা	☐

১৪। খামারের জীব নিরাপত্তার ধরণ?

উত্তম	☐
মধ্যম	☐
নিম্ন মানের	☐
