# Supplementary material for: Bovine tuberculosis prevalence and risk factors in selected districts of Bangladesh
Source: PLoS One. 2020 Nov 10;15(11):e0241717. doi: 10.1371/journal.pone.0241717 (PMC7654795; doi:10.1371/journal.pone.0241717)
Supplement: S3 Questionnaire — (DOCX) [file pone.0241717.s006.docx]

**S3 Questionnaire**. Survey interview questionnaire for possible risk factors of bTB responsible for animal level infection in urban and periurban areas of some selected district of Bangladesh

| 1. Animal ID number: | |  |  |  |  |  |  |
| --- | --- | --- | --- | --- | --- | --- | --- |
|  |  |  |  |  |  |  |  |
| 2. Sex of the animal | |  |  | 2. Source of the animal? | |  |  |
|  |  |  |  |  |  |  |  |
|  | Male |  |  |  | Farm |  |  |
|  |  |  |  |  |  |  |  |
|  | Female |  |  |  | Bought |  |  |
|  |  |  |  |  |  |  |  |
| 3. Age of the animal (in months) | | |  | 4. Breed of the animal | |  |  |
|  | ……………….months |  |  |  |  |  |  |
|  |  |  |  | Frisian Cross |  |  |  |
|  |  |  |  |  |  |  |  |
|  |  |  |  | Shahiwal/Sindhi Cross | |  |  |
|  |  |  |  |  |  |  |  |
|  |  |  |  | Other cross ( Brahma/Jersey cross) | | |  |
|  |  |  |  |  |  |  |  |
|  |  |  |  | Native or indigenous | |  |  |
|  |  |  |  |  |  |  |  |
| 5. Tentative body weight in (Kg) | | |  | 6. Milking status in female animal | | |  |
|  |  |  |  |  |  |  |  |
|  | 1-100 Kg |  |  |  | Yes |  |  |
|  |  |  |  |  |  |  |  |
|  | 100-200 Kg |  |  |  | No |  |  |
|  |  |  |  |  |  |  |  |
|  | 200-400 Kg |  |  | 7. If yes, daily milk production per cow | | |  |
|  |  |  |  |  |  |  |  |
|  | 400-500 Kg |  |  |  |  | liter |  |
|  |  |  |  |  |  |  |  |
|  | >500 Kg |  |  |  |  |  |  |
| 8. Stage of lactation | |  |  | 9. Status of pregnancy? | |  |  |
|  |  |  |  |  |  |  |  |
|  | 1st stage |  |  | Yes |  |  |  |
|  |  |  |  |  |  |  |  |
|  | Second stage |  |  | No |  |  |  |
|  |  |  |  |  |  |  |  |
|  | 3rd stage |  |  |  |  |  |  |
|  |  |  |  |  |  |  |  |
| 10. Number of parity? | |  | |  |  |  |  |
|  |  |  |  |  |  |  |  |
| 11. Body condition score? | | |  |  |  |  |  |
|  |  |  |  |  |  |  |  |
|  | Bad (BCS: 0-3) |  |  |  |  |  |  |
|  |  |  |  |  |  |  |  |
|  | Good (BCS: >6) |  |  |  |  |  |  |
|  |  |  |  |  |  |  |  |
|  | Medium (BCS: 4-6) |  |  |  |  |  |  |
|  |  |  |  |  |  |  |  |
