# Supplementary material for: Bovine tuberculosis prevalence and risk factors in selected districts of Bangladesh
Source: PLoS One. 2020 Nov 10;15(11):e0241717. doi: 10.1371/journal.pone.0241717 (PMC7654795; doi:10.1371/journal.pone.0241717)
Supplement: S4 Questionnaire — (PDF) [file pone.0241717.s007.pdf]

এস ৬ প্রশ্নাবলী। পশু পর্যায়ে টিবি বা যক্ষা সংক্রমণ ঝুঁকি বিষয়ক সার্ভে প্রশ্নমালা

(সাক্ষাৎকার গ্রহণকারী কর্তৃক পূরনকৃত)

১। গবাদিপশুর ট্যাগ বা আইডি নং

২। পশুর লিংগ

পুঃ

স্ত্রী

৩। পশুর উৎস ?

খামার

ক্রয়কৃত

৩। পশুর আনুমানিক বয়স

..................মাস

৫. গবাদিপশুর জাত

ফ্রিজিয়ান ক্রস

শাহীওয়াল/সিন্ধি ক্রস

অন্যান্য ক্রস (ব্রাহামা/জারসী)

দেশী

৫। আনুমানিক ওজন (কেজি)

<১০০ কেজি

১০০-২০০ কেজি

২০০-৪০০ কেজি

৪০০-৫০০ কেজি

>৫০০ কেজি

৬। গাভী দুধ উৎপাদন করছে কিঃ

হ্যাঁ (দুগ্ধবতী)

না (শুষ্ক)

৭। হ্যা হলে দৈনিক দুধ উৎপাদনের পরিমান

লিটার

৮। দুধ উৎপাদনের পর্যায়

১ম পর্যায়

২য় পর্যায়

৩য় পর্যায়

৮। গর্ববতী কিনা?

হ্যাঁ

না

১০। এ পর্যন্ত কত বার বাচ্চা দিয়েছে।

১১। শারিরিক অবস্থার স্কোর (বিসিএস)

খারাপ (বিসিএসঃ ০-৩) ☐

ভাল (বিসিএসঃ >৬) ☐

মধ্যম (বিসিএসঃ ৪-৬) ☐
